# HDAC2 hyperexpression alters hippocampal neuronal transcription and microglial activity in neuroinflammation-induced cognitive dysfunction

**DOI:** 10.1186/s12974-019-1640-z

**Published:** 2019-12-03

**Authors:** Xiao-Yu Sun, Teng Zheng, Xiu Yang, Le Liu, Shen-Shen Gao, Han-Bing Xu, Yu-Tong Song, Kun Tong, Li Yang, Ya Gao, Tong Wu, Jing-Ru Hao, Chen Lu, Tao Ma, Can Gao

**Affiliations:** 1 Jiangsu Province Key Laboratory of Anesthesiology, Xuzhou Medical University, 209 Tongshan Road, Xuzhou, 221004 Jiangsu China; 2 Jiangsu Province Key Laboratory of Anesthesia and Analgesia Application, Xuzhou Medical University, Xuzhou, 221004 Jiangsu China; 3 Department of Anesthesiology, the First Affiliated Hospital of Soochow University, Suzhou, 215006 Jiangsu China

**Keywords:** Activated microglia, Cognitive impairment, HDAC2, Isoflurane, Lipopolysaccharide, Neuroinflammation

## Abstract

**Background:**

Inflammation can induce cognitive dysfunction in patients who undergo surgery. Previous studies have demonstrated that both acute peripheral inflammation and anaesthetic insults, especially isoflurane (ISO), are risk factors for memory impairment. Few studies are currently investigating the role of ISO under acute peri-inflammatory conditions, and it is difficult to predict whether ISO can aggravate inflammation-induced cognitive deficits. HDACs, which are essential for learning, participate in the deacetylation of lysine residues and the regulation of gene transcription. However, the cell-specific mechanism of HDACs in inflammation-induced cognitive impairment remains unknown.

**Methods:**

Three-month-old C57BL/6 mice were treated with single versus combined exposure to LPS injected intraperitoneally (i.p.) to simulate acute abdominal inflammation and isoflurane to investigate the role of anaesthesia and acute peripheral inflammation in cognitive impairment. Behavioural tests, Western blotting, ELISA, immunofluorescence, qRT-PCR, and ChIP assays were performed to detect memory, the expressions of inflammatory cytokines, HDAC2, BDNF, c-Fos, acetyl-H3, microglial activity, *Bdnf* mRNA, *c-fos* mRNA, and *Bdnf* and *c-fos* transcription in the hippocampus.

**Results:**

LPS, but not isoflurane, induced neuroinflammation-induced memory impairment and reduced histone acetylation by upregulating histone deacetylase 2 (HDAC2) in dorsal hippocampal CaMKII^+^ neurons. The hyperexpression of HDAC2 in neurons was mediated by the activation of microglia. The decreased level of histone acetylation suppressed the transcription of *Bdnf* and *c-fos* and the expressions of BDNF and c-Fos, which subsequently impaired memory. The adeno-associated virus Sh*Hdac2*, which suppresses *Hdac2* after injection into the dorsal hippocampus, reversed microglial activation, hippocampal glutamatergic BDNF and c-Fos expressions, and memory deficits.

**Conclusions:**

Reversing HDAC2 in hippocampal CaMKII^+^ neurons exert a neuroprotective effect against neuroinflammation-induced memory deficits.

## Introduction

Peri-inflammatory conditions in the abdomen induce pathological and molecular changes in the brain [1-4]. Brain uptake of circulating lipopolysaccharide (LPS) is not sufficient to cross the brain-blood barrier (BBB) that most effects of peripherally administered LPS are mediated through LPS receptors located outside the BBB [5]. Pro-inflammatory cytokines released in the periphery are involved in peripheral immune system-to-brain communication by activating resident microglia in the brain and subsequently increasing pro-inflammatory factors including TNF-α and IL-1 [6–8]. As reported previously, a variety of patients undergo surgery for abdominal inflammation, and some patients who undergo surgery suffer from surgery-induced cognitive impairment, leading to prolonged hospitalization and an increased mortality rate [9–12]. The systemic inflammation induced by surgery triggers hippocampal inflammation and induces memory impairment [13]. In addition, accumulating evidence indicates that general anaesthesia may exacerbate this process [14–16]. A previous study revealed a selective but significant induction of inflammation in children after isoflurane (ISO) anaesthesia without surgical stress [17]. ISO may increase neuroinflammation, leading to sickness behaviour [18–20]. However, few studies are currently investigating the role of ISO under acute peri-inflammatory conditions, and it is difficult to predict whether ISO can aggravate inflammation-induced cognitive deficits. The molecular mechanisms underlying this cognitive impairment are still unknown.

Epigenetic regulations play an important role in cognition. The activities of histone acetyltransferases (HATs) and histone deacetylases (HDACs) are involved in histone modification, leading to varying effects on transcription [21–24]. Specific sites on histones, such as H3K9, H3K14, and H3K8, can be acetylated by HATs, which are recognized as coactivators of transcription factors. In addition, HDACs play a role in the deacetylation of lysine residues, and this process is highly reversible [25]. HDACs activities can stabilize the local chromatin structure, which predominantly acts as transcriptional repressors [26, 27]. HDACs have been widely recognized as playing significant roles in learning and memory formation. Although the hippocampus is involved in multiple aspects of learning and memory, the mechanism by which hippocampal HDACs participate in the regulation of neuroinflammation-induced cognitive deficits remains unclear.

In this study, we used an animal model with a single exposure to LPS (1 mg/kg, i.p.) and 1.5% ISO inhalation for 4 h to investigate the effects of anaesthesia and acute peripheral inflammation on cognitive impairments. We aimed to investigate the role of HDACs in our animal model and to explore an approach for inflammation-induced cognitive deficits. Our study demonstrated that the neuroinflammation induced by LPS rather than isoflurane increased the expression of HDAC2 in hippocampal CaMKII^+^ neurons via microglial activation. Hyperexpression of HDAC2 decreased the transcription of *Bdnf* and *c-fos*, which subsequently impaired memory. The suppression of HDAC2 in CaMKII^+^ neurons reversed inflammatory cytokines and microglial activation. Accordingly, suppressing HDAC2 in the hippocampus may be an approach for inflammation-induced cognitive deficits.

## Materials and methods

### Animals

C57BL/6 male mice (3 months old, weighing 21–25 g) were obtained from Xuzhou Medical University (SYXK 2016-0028). All the mice were housed under standard conditions with four to six mice per cage and were kept in a room (22 ± 2 °C) maintained on a 12 h/12 h dark/light cycle (08:00 A.M.–08:00 P.M.) with ad libitum access to food and water. The animals were acclimatized for 7 days before the experiments and were group-housed with the same cage mates throughout the acclimation and experiment. All animal experiments were performed in accordance with the Use of Laboratory Animals and the requirements of the Animal Ethics Committee of Xuzhou Medical University, Jiangsu, China. All experiments were conducted in compliance with the Animal Research Reporting In Vivo Experiments (ARRIVE) guidelines.

### Animal experimental groups

In the first set of experiments, the mice were randomly divided into four groups: the control group (Con), the ISO group (ISO), the LPS group (LPS) and the ISO plus LPS group (LPS + ISO). The mice in the LPS group received an intraperitoneal injection of LPS (1 mg/kg, Sigma-Aldrich, USA). As previously reported, 0.25, 0.33, 1 and 1.5 mg/kg LPS (i.p.) will elicit inflammatory cytokine responses in the brain, which subsequently induce sickness behaviour in mice. Memory impairment induced by the 1- and 1.5-mg/kg doses of LPS elicited cognitive dysfunction lasting more than 2 days [28–30]. A LPS dose of 1 mg/kg was selected in our experiment according to the behaviour results. The mice in the ISO group were placed in a crystal chamber prefilled with 1.5% ISO in 100% oxygen for 4 h. A 1.3 minimum alveolar concentration (MAC) of ISO (1.5%) was chosen to prevent a motor response in 99% of subjects in response to a surgical stimulus. Previous studies demonstrate that the mean operative time in abdominal surgery, such as laparoscopic general surgery, is 162.1 min and that an operation time > 4 h is a potential predictor of prolonged postoperative ileus after major abdominal surgery [31, 32]. The mice in the LPS + ISO group were injected with LPS after ISO anaesthesia exposure for 4 h.

In the second set of experiments, the mice were randomly divided into four groups: the control group (Con), LPS group (LPS), LPS plus pAAV-ZsGreen-ShRNA-mHDAC2 group (LPS + ShHdac2), and LPS plus pAAV-ZsGreen-ShRNA-mScramble group (LPS + ShSc). The mice in the LPS group received an intraperitoneal injection of LPS (1 mg/kg). The mice in the LPS + ShHdac2 group received bilateral microinjection of pAAV-ZsGreen-ShRNA-mHDAC2 into the dorsal region of the hippocampus (1 μl per site) and intraperitoneal injection of LPS (1 mg/kg). The mice in the LPS + ShSc group received bilateral microinjection of pAAV-ZsGreen-ShRNA-mScramble into the dorsal region of the hippocampus (1 μl per site) and an intraperitoneal injection of LPS (1 mg/kg).

A detailed timeline for each in vivo experiment is presented in Additional file 1: Figure S1a.

### Construction of adeno-associated viruses and stereotaxic injections

An adeno-associated virus (AAV; serotype 9) that reduced *Hdac2* expression was constructed by Biowit Company (Shenzhen, China). The primer sets for the mice were as follows: *Hdac2*-1: 5′-CCCAATGAGTTGCCATATAAT-3′ and *Hdac2*-2: 5′-CGAGCATCAGACAAACGGATA-3′. Mice were anaesthetized by an intraperitoneal injection of Avertin (isoamyl alcohol, 250 mg/kg). The mice were fixed in a stereotaxic frame, and the AAV vectors were stereotaxically injected bilaterally into the dorsal region of the hippocampus (1 μl per site) at the following coordinates (Additional file 2: Figure S2 a, b), as we described previously: a/p, − 1.5; m/l, ± 1.0; and d/v, – 2.0 [33]. Behavioural tests were carried out 3 weeks after virus injection.

### Open-field test

All behavioural procedures were performed during the light phase of the light/dark cycle between 10:00 A.M. and 4:00 P.M. in an acoustically isolated room. An open-field apparatus was positioned in a dimly lit room and consisted of a black Plexiglas chamber with a white floor (45 cm × 45 cm), as we described previously [34]. Each mouse was placed in the centre of the area and allowed to explore the whole field for 5 min, with the movements of the mice automatically recorded by Anymaze software. The total distance and the time spent in the centre area were measured as the parameters of anxiolytic behaviour.

### Fear conditioning

Both contextual and tone-dependent fear conditioning were performed with an automated system (Med Associates, Inc., USA). The conditioning consisted of a single exposure to a context (3 min) followed by a 30 s tone (10 kHz; 75 dB SPL) and a foot shock (2 s; 0.7 mA; constant current), as we described previously [35, 36]. Context-dependent freezing was measured 24 h later every 10 s over 180 s by two observers who were unaware of the experimental conditions, and the results are expressed as the percentage of freezing frequency. Freezing in response to the tone was similarly scored every 5 s in a novel context during a 30-s exposure.

### Novel object recognition task

During the training phase, each mouse explored two identical “sample” objects, object 1 (O1) and object 2 (O2), for 10 min. The mouse was then returned to its home cage for a retention period of 1 h before being reintroduced to the training context and presented with a previous sample object, object 1, and a novel object, object 3 (O3), for 5 min. Movement and interaction with the objects were recorded automatically by Anymaze software. Exploratory behaviour was defined as sniffing, licking, or touching the object while facing the object. Memory was assessed by measuring the discrimination ratio (i.e., the ratio of the time spent exploring the novel object to the time spent exploring both objects). The total interaction time with both objects was compared with determine whether the treatments affected locomotor activity and/or exploration.

### Enzyme-linked immunosorbent assay

Concentrations of interleukin-1β (IL-1β), interleukin-6 (IL-6), and tumour necrosis factor α (TNF-α) were examined using enzyme-linked immunosorbent assay (ELISA) kits following the manufacturer’s instructions (R&D Systems, UK). For measurement of protein concentrations in plasma, whole blood was collected in heparinized tubes. After removing the cells by centrifugation in a refrigerated centrifuge, the supernatant (plasma) was used for ELISA measurements. Absorbance was recorded with a spectrophotometer at a wavelength of 450 nm. The concentrations of IL-1β, IL-6, and TNF-α were calculated according to a standard curve and are presented as pg/mg protein.

### Immunoblot analysis

To measure the protein concentrations in the hippocampus, we homogenized the tissue in RIPA lysis buffer supplemented with a cocktail of protease and phosphatase inhibitors (Applygen, China) on ice. After centrifugation, the supernatant protein concentration was determined with a bicinchoninic acid (BCA) protein assay reagent kit (Thermo Pierce, USA). After determination of the protein concentration, protein samples were separated by SDS-PAGE and subsequently transferred to PVDF membranes (Bio-Rad, USA). Membranes were blocked with 5% nonfat milk in TBST (0.1% Tween-20 in TBS) and then incubated at 4 °C overnight with primary antibodies: HDAC2 (1:2000, Cell Signaling Technologies, USA), HDAC3 (1:2000, Santa Cruz, USA), HDAC4 (1:2000, Cell Signaling Technologies, USA), HDAC6 (1:4000, Merck Millipore, USA), Acetyl-H3 (1:2000, Merck Millipore, USA), Acetyl-H3K9 (1:2000, Merck Millipore, USA), Acetyl-H3K14 (1:2000, Merck Millipore, USA), BDNF (1:1000, Abcam, UK), c-Fos (1:2000, Merck Millipore, USA), and β-actin (1:2000, Santa Cruz, USA). After being rinsed with TBST, the membranes were incubated with horseradish peroxidase-conjugated secondary antibodies (1:1000, Beyotime Institute of Biotechnology, China). Protein bands were visualized with an ECL detection system (Beyotime Institute of Biotechnology, China) and quantified with ImageJ software (NIH).

### Immunofluorescence staining

Mice were perfused transcardially via the left ventricle with phosphate-buffered saline (PBS; pH 7.4), followed by 4% paraformaldehyde in PBS (pH 7.4). The brains were harvested and submerged in 4% paraformaldehyde at 4 °C overnight, dehydrated in 30% sucrose for 3 days, embedded with OCT (Leica, Germany), and stored at − 80 °C. Coronal sections (40 μm) were sliced on a cryostat (Leica CM1950, Germany). The slices were rinsed with PBS and then incubated with 10% normal donkey serum in PBST for 1 h at room temperature followed by primary antibodies dissolved in 5% normal donkey serum for 24 h at 4 °C: Iba1 (1:400, Abcam, UK), BDNF, (1:400, Abcam, UK), c-Fos (1:400, Merck Millipore, USA), HDAC2 (1:1000, Santa Cruz, USA), acetyl-H3 (1:500, Merck Millipore, USA), CaMKIIα (1:400, Santa Cruz, USA), and GFAP (1:400, Abcam, UK). The sections were washed with PBST and incubated at room temperature for 1 h with secondary antibodies: donkey anti-rabbit (1:200; Thermo Fisher Scientific, USA), donkey anti-mouse (1:200; Thermo Fisher Scientific, USA), and donkey anti-goat (1:200; Thermo Fisher Scientific, USA). Then, the sections were mounted with DAPI Fluoromount-G (Southern Biotech, USA). An FV-1000 confocal fluorescence microscope (Olympus, Japan) was used to acquire images. Images were quantified by ImageJ software.

### Cell cultures and treatments

For BV2 microglia culture, the medium was changed every other day using a 1:1 mixture of fresh DMEM (Gibco, USA) containing 10% foetal bovine serum (FBS). The cells were kept at 37 °C in a humidified incubator with 5% CO_2_/95% air and used for experiments after treatment. BV2 cells were treated with LPS (1 μg/ml) or left untreated for 12 h, and then the medium was replaced with identical medium. After 12 h, the BV2 supernatant was transferred to the primary neuron culture after centrifugation at 1000 rpm for 3 min.

For primary neuron culture, the hippocampi from embryonic day 17-19 C57/BL mice were isolated and dissociated with trypsin. Cells were plated in 6-well culture plates coated with poly-D-lysine and grown in NeuroBasal medium with B-27 (Invitrogen; 17504). One half of the medium was replaced with identical medium every 4 days. Cultures were kept at 37 °C in a humidified incubator with 5% CO_2_/95% air and used for experiments after 18–21 days in vitro. The medium from BV2 microglia treated with LPS (1 μg/ml), U0126 or left untreated was transferred to the primary neurons for 12 h. The cultures were collected in cold homogenization buffer (in mM: 50 MOPS (pH 7.4), 320 sucrose, 100 KCl, 0.5 MgCl_2_, 0.2 DTT, 50 NaF, 20 NaPPi, 20 β-glycerophosphate, 1 EDTA, 1 EGTA, 1 PNPP-Na, 1 Na_3_VO_4_, 0.5 PMSF, 10 μg/ml leupeptin, 10 μg/ml aprotinin, 10 μg/ml pepstatin A, and 100 μg/ml benzamidine).

### Quantitative real-time reverse transcription PCR

Total RNA was extracted from the dorsal hippocampus using a Universe RNA extraction kit according to the manufacturer’s protocol (Takara, Japan). cDNA was synthesized from 400 ng of RNA with the Prime Script RT Reagent Kit (Takara, Japan) and then subjected to real-time PCR to measure *Bdnf*, *c-fos*, *Il-1β*, *Tnf-α*, *Il-6*, and *Gapdh* levels using SYBR Green PCR master mix (Takara, Japan). PCR assays were conducted in a LightCycler 480 real-time PCR system (Roche, Switzerland), and the data are shown as fold changes. The primer sequences for *Bdnf*, *c-fos*, *Il-1β*, *Tnf-α*, *Il-6*, and *Gapdh* were as follows:

*Bdnf*, *F*: *5*′*-*CATAAGGACGCGGACTTGTACA-3′ and R: 5′-AGACATGTTTGCGGCATCCA-3′; *c-fos*, F: 5′-GAAAGCCTGGGGCGTAGAGT-3′ and R: 5′-CCTCAGCTGGCGCCTTTAT-3′; *Il-1β*, F: 5′-CAACCAACAAGTGATATTCTCCATG-3′ and R: 5′-GATCCACACTCTCCAGCTGCA-3′; *Il-6*, F: 5′- CCTGGAGTTTGTGAAGAACAACT-3′ and R: 5′-GGAAGTTGGGGTAGGAAGGA-3′; *Tnf-α*, F: 5′-CTGTAGCCCACGTCGTAGCA-3′ and R: 5′-CGGCAGAGAGGAGGTTGACT-3′; and *Gapdh*, F: 5′-TGAAGGTCGGAGTCAACGGATTTGGT-3′ and R: 5′-CATGTGGGCCATGAGGTCCACCAC-3′.

### Chromatin immunoprecipitation

Chromatin immunoprecipitation (ChIP) assays were performed with an anti-acetyl-H3 antibody using Magna one-day chromatin immunoprecipitation kits following the manufacturer’s instructions (Merck Millipore, USA). PCR analysis was conducted in a real-time PCR system as previously described, and promoter enrichment was analysed using qPCR of ChIP DNA versus input. The primer sequences for the *Bdnf* and *c-fos* promoters were as follows:

*Bdnf* VI, F: 5′-AAACCAGGGGAGAAAGATTTG-3′ and R: 5′-GGAGGAAGCGAGTGTGAGTC-3′; c-fos, F: 5′-CTCTCGGCCGACTTGTTTCT-3′ and R: 5′-GCGACTCTTTGCTCGAGACT-3′.

### Data analysis and statistics

Data are presented as the mean ± S.E.M. and were analysed with Statistical Package for the Social Sciences (SPSS; version 17.0, IL, USA) software. In the first set of experiments (Con, ISO, LPS, and LPS + ISO), the difference among the four groups was determined by two-way analysis of variance followed by Bonferroni’s post hoc test. ISO and LPS were considered two independent factors. One-way analysis of variance (ANOVA) followed by the Student-Newman-Keuls least-significant difference test (for equal variances) or the Dunnett T3 (for unequal variances) test was used to compare the results in the second set (Con, LPS, LPS + ShHdac2, and LPS + ShSc) of behavioural and other results, as described in a previous study [37]. Paired *t* tests were used for analyses between two pairs of time points in the same treatment group. Pairwise comparisons between groups were performed with an independent *t* test. Differences were considered significant when *P* < 0.05.

## Results

### LPS but not ISO inhalation induces cognitive impairments and HDAC2 upregulation in adult mice

**Fig. 1:**
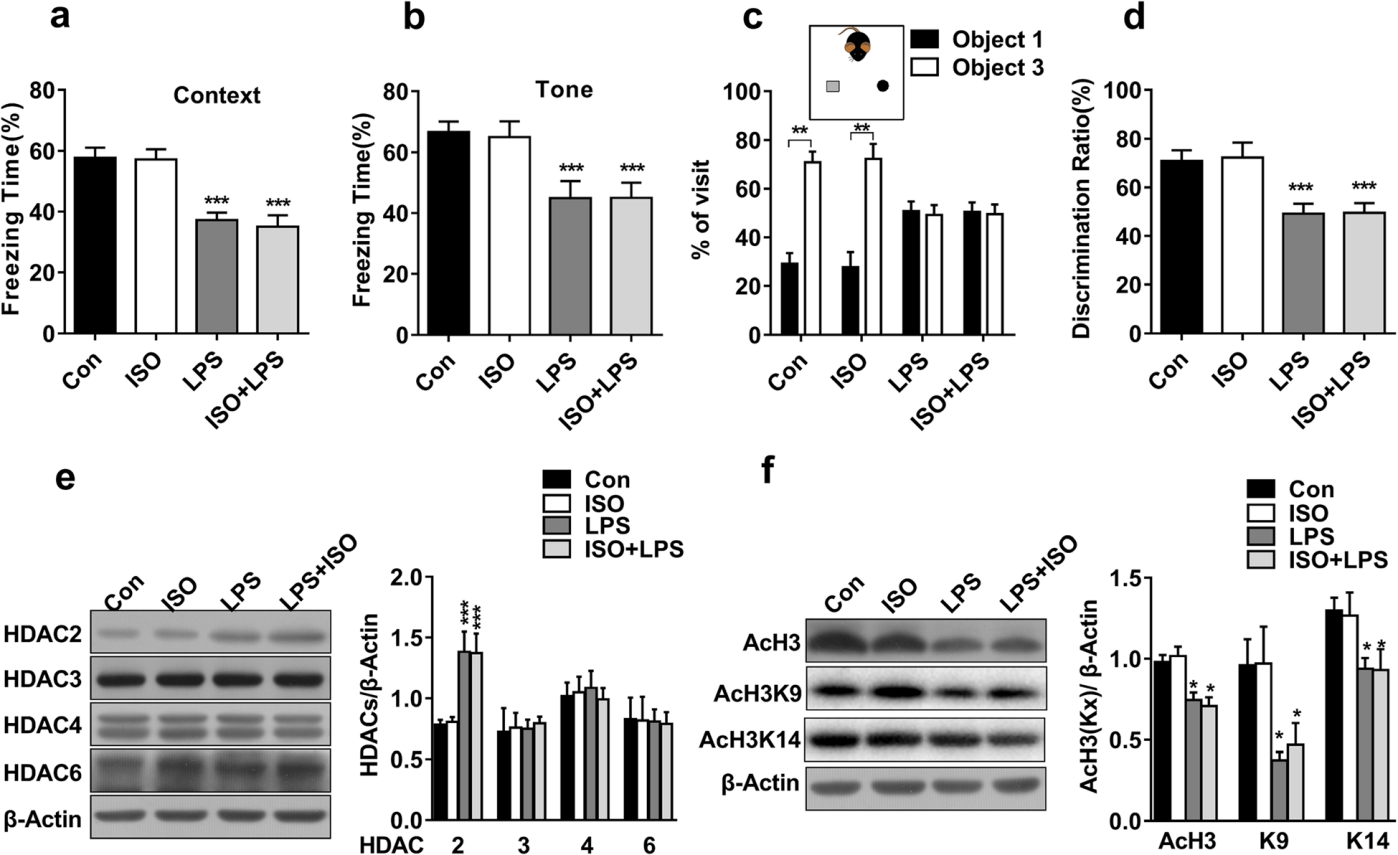
LPS, not ISO, induced cognitive impairments and HDAC2 upregulation in mice. **a**, **b** The mice in the LPS and ISO + LPS groups but not those in the ISO group showed significantly impaired context-dependent fear memory and tone-dependent fear memory (*n* = 10). **c** Impaired object discrimination was observed in the LPS and ISO + LPS groups but not in the ISO group, as revealed by similar percentages for visiting familiar object 1 and novel object 3. **d** No preference for novel object 3 was observed in the LPS or ISO + LPS group compared with the control group (*n* = 8). **e** The expression of HDAC2, but not HDAC3, HDAC4 or HDAC6, was increased in the LPS and LPS + ISO groups but not in the ISO group (*n* = 4). **f** The expressions of acetyl-H3, acetyl-H3K9, and acetyl-H3K14 were increased in the LPS and LPS + ISO groups but not in the ISO group (*n* = 4). Data are presented as the mean ± S.E.M. ^*^P < 0.05; ^**^P < 0.01; and ^***^P < 0.001

Emotional disturbances and memory deficits have been reported in both inflammation and post-surgery patients [38–40]. We first tested whether exposure to ISO or LPS could induce anxiety-like behaviour in mice. In an open-field test, the total distance travelled (Additional file 1: Figure S1b) and the time spent in the centre area (Additional file 1: Figure S1c) showed no significant differences among all groups. Next, we investigated the effects of ISO and LPS on learning and memory. As shown in fear conditioning tests, compared with the control group, LPS exposure but not ISO exposure led to decreased freezing times in contextual (*F*_(1,36)_ = 42.694, *P* < 0.001; Fig. 1a) and cued (*F*_(1,36)_ = 18.090, *P <* 0.001; Fig. 1b) fear memory. However, the interaction between LPS and ISO was not statistically significant in contextual (*F*_(1.36)_ = 0.061, *P* = 0.807) or cued (*F*(_1.36)_ = 0.029, *P* = 0.866) fear memory. ISO exposure did not aggravate the memory deficits caused by LPS exposure. In a novel object recognition task, all groups showed no significant preference in the novel objection recognition test during training (Additional file 1: Figure S1d-e). During testing, the mice in the LPS group and LPS + ISO group did not spend more time on novel object than on familiar object, whereas the mice in the Con group and ISO group spent more time exploring novel object (*t*_(7, 0.05)_ = 4.732, *P* < 0.01; Fig. 1c). The discrimination ratio difference was significant in the LPS group and the LPS + ISO group compared with the Con group (*F*_(1,28)_ = 21.980, *P* < 0.001; Fig. 1d). There was no significant interaction between LPS and ISO (*F*_(1.28)_ = 0.012, *P* = 0.914). These results showed that the memory performance impairment induced by exposure to LPS was not aggravated by ISO exposure. As reported previously, LPS elicits an inflammatory cytokine response in the brain, resulting in transient memory deficits in mice. Our results also defined a single exposure to the volatile inhaled anaesthetic ISO as safe in adult mice.

We then examined the effects of LPS and ISO on the expressions of HDACs and acetylated histones. The level of HDAC2 was increased significantly when the mice were exposed to LPS but not when they were exposed to ISO (*F*_(1,12)_ = 23.864, *P* < 0.001). The interaction between LPS and ISO was not statistically significant (*F*_(1.12)_ = 0.022, *P* = 0.886). However, there were no significant changes in the expressions of HDAC3, HDAC4 and HDAC6 among the four groups (Fig. 1e). The levels of acetyl-H3 were decreased in the LPS group and LPS + ISO group, whereas no change was found in the ISO group (*F*_(1,12)_ = 28.973, *P* < 0.001). We further detected two significant acetylation sites, H3K9 and H3K14, which have been implicated in learning and memory as well as the excitement of synaptic plasticity [41]. The hippocampal acetyl-H3K9 (*F*_(1,12)_ = 11.27, *P* < 0.01) and acetyl-H3K14 levels (*F*_(1,12)_ = 10.28, *P* < 0.01; Fig. 1f) were decreased in the LPS group and the LPS + ISO group compared with the Con group, whereas no change was found in the ISO group. The analysis also showed no significant ISO and LPS interactions in the expression of acetyl-H3K9 (*F*_(1.12)_ = 0.007, *P* = 0.79) and acetyl-H3K14 (*F*_(1,12)_ = 0.01, *P* = 0.914). In the following experiments, we focused on the HDAC2-mediated mechanism of LPS-induced cognitive impairment.

### The HDAC2 is distributed in glutamatergic neurons in the hippocampus

**Fig. 2:**
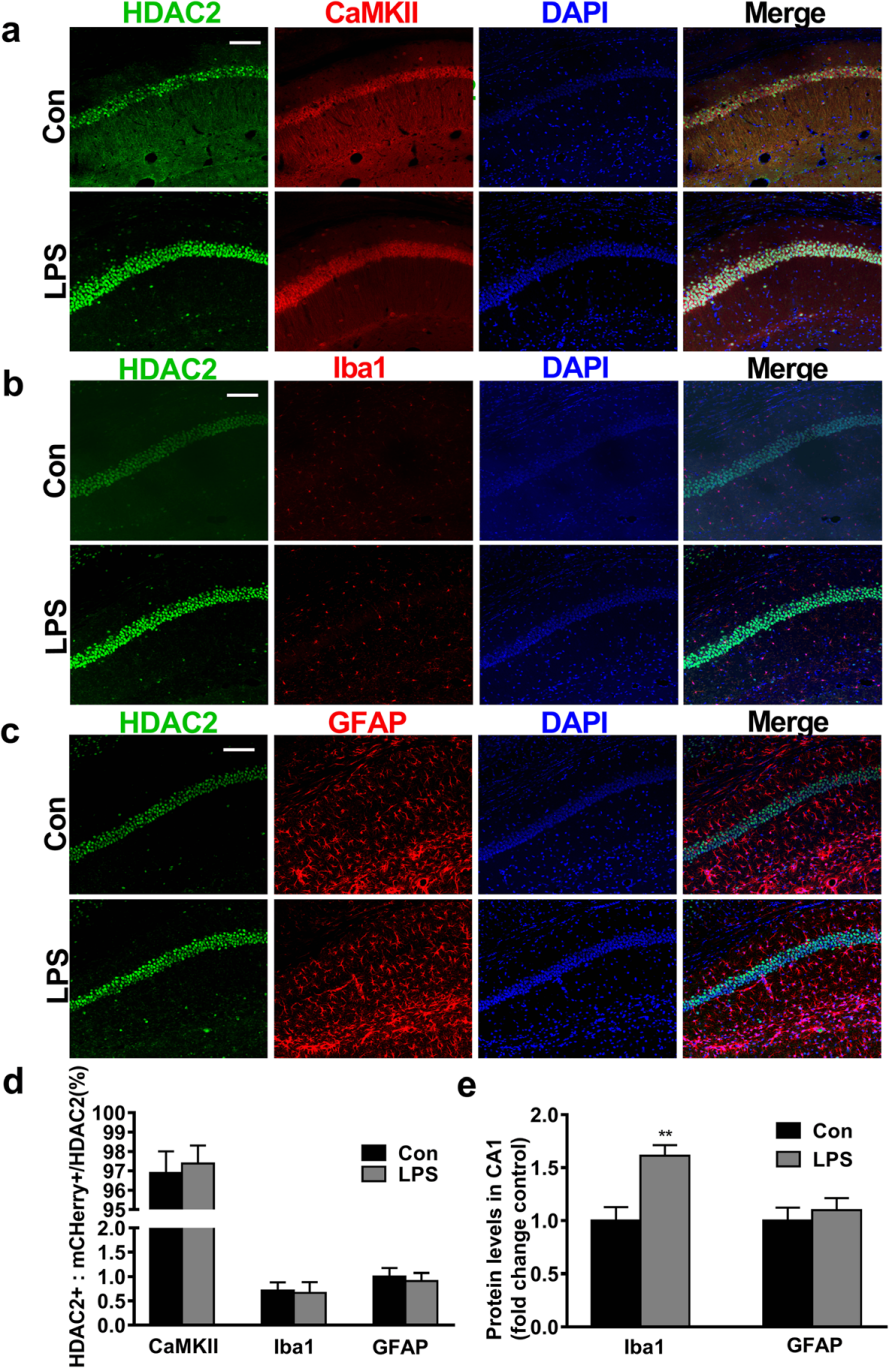
The majority of the HDAC2 protein was colocalized with CaMKII^+^ neurons, and microglia were activated after LPS administration. **a–c** Representative images of coimmunostaining for HDAC2 and CaMKII, Iba1 or GFAP in the hippocampal CA1 region are shown (*n* = 3–4 sections from 4 mice, scale bar = 100 μm). **d** The majority of HDAC2 was colocalized with CaMKII rather than Iba1 or GFAP (*n* = 4). **e** The expression of Iba1 was significantly increased in the LPS group (*n* = 4). Data are presented as the mean ± S.E.M. ^*^P < 0.05 and ^**^P < 0.01

A previous study reported a significant upregulation of HDAC2 expression in laser-captured nigral microglia in Parkinson’s disease [42]. However, the cell-specific HDAC2 mechanism underlying neuroinflammation-induced memory deficits remains unknown. To this end, we labelled HDAC2 and calcium/calmodulin-dependent protein kinases (CaMKII), ionized calcium-binding adaptor molecule1 (Iba1) or glial fibrillary acidic protein (GFAP) by immunostaining. CaMKII, Iba1, and GFAP are biomarkers of glutamatergic neurons, microglia and astrocytes, respectively [43–45]. Our results showed that less than 1% of the detected HDAC2 protein was expressed on astrocytes or microglia. The majority of the detected HDAC2 protein (> 96%) was colocalized with CaMKII in the hippocampus (Fig. 2a–d), which indicated that HDAC2 dominantly regulated histone acetylation in hippocampal glutamatergic neurons. Compared with the control treatment, LPS exposure increased the expression of microglial Iba1 (*t*_(3, 0.05)_ = 3.767, *P* < 0.01), whereas the expression of GFAP remained unchanged (*t*_(3, 0.05)_ = 0.5912, *P* = 0.576; Fig. 2e). The expression of Iba1 showed no significant change in the ISO group compared with the Con group (Additional file 3: Figure S3a-b). It was previously reported that during neuroinflammation, astrocytes undergo morphological changes that are not strictly dependent on GFAP protein level [46]. We performed a microscopy analysis and found that the cellular morphology of astrocyte was nearly unaffected in the LPS group (Additional file 4: Figure S4).

### The hyperexpression of HDAC2 in neurons is mediated by microglial activation induced by LPS

**Fig. 3:**
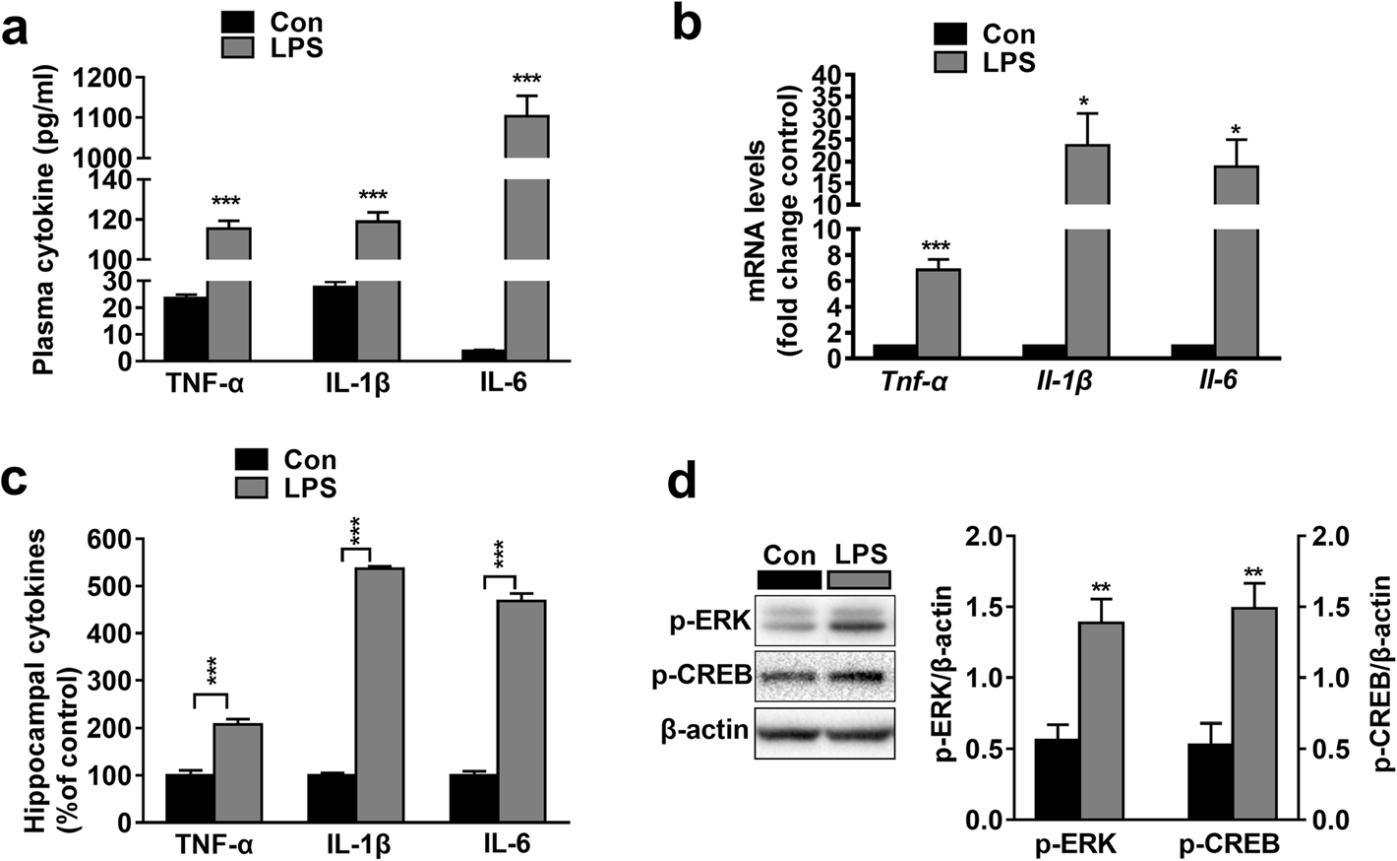
The expressions of peripheral and central pro-inflammatory cytokines were increased in LPS mice. **a** The levels of TNF-α, IL-1β, and IL-6 in plasma samples were increased in the LPS group (***n*** = 4). **b** The mRNA levels of *Tnf-α*, *Il-1β* and *Il-6* in samples from the hippocampus were increased in the LPS group (***n*** = 4). **c** The protein levels of TNF-α, IL-1β, and IL-6 in samples from the hippocampus were increased in the LPS group. **d** The expression of p-ERK and p-CREB in the hippocampal CA1 region was increased in the LPS group (***n*** = 4). Data are presented as the mean ± S.E.M. ^*^P < 0.05; ^**^P < 0.01; and ^***^P < 0.001

Microglia are considered the innate immune cells of the central nervous system (CNS). Upon activation, microglia release pro-inflammatory cytokines and cytotoxic compounds that disrupt homeostatic processes and neuronal functions [47, 48]. Our data showed that LPS induced the accumulation of TNF-α (*t*_(3, 0.05)_ = 22.04, *P* < 0.001), IL-1β (*t*_(3, 0.05)_ = 17.97, *P* < 0.001) and IL-6 (*t*_(3, 0.05)_ = 21.88, *P* < 0.001; Fig. 3a) in plasma. The mRNA expressions of *Tnf-α* (*t*_(3, 0.05)_ = 7.276, *P* < 0.001), *Il-1β* (*t*_(3, 0.05)_ = 3.045, *P* < 0.001) and *Il-6* (*t*_(3, 0.05)_ = 2.855, *P* < 0.05; Fig. 3b) as well as the protein expressions of TNF-α (*t*_(3, 0.05)_ = 7.118, *P* < 0.001), IL-1β (*t*_(3, 0.05)_ = 62.88, *P* < 0.001), and IL-6 (*t*_(3, 0.05)_ = 20.18, *P* < 0.001; Fig. 3c) in the hippocampus of the LPS group were also higher than those in the Con group. The levels of plasma and hippocampal pro-inflammatory cytokines remained unchanged (Additional file 3: Figure S3c-d). Given that HDAC2 decreases the levels of acetylated histones and suppresses transcription, it is implied that cytokine released by microglia is elevated through another mechanism. We examined the expression of p-ERK1/2 and p-CREB, which positively regulate transcription [49, 50]. Interestingly, we found that LPS increased hippocampal p-ERK1/2 (*t*_(3, 0.05)_ = 4.107, *P* < 0.01) and p-CREB levels (*t*_(3, 0.05)_ = 4.154, *P* < 0.01; Fig. 3d), which indicated that LPS increased pro-inflammatory cytokine expression and microglial activity through a MAPK pathway.

**Fig. 4:**
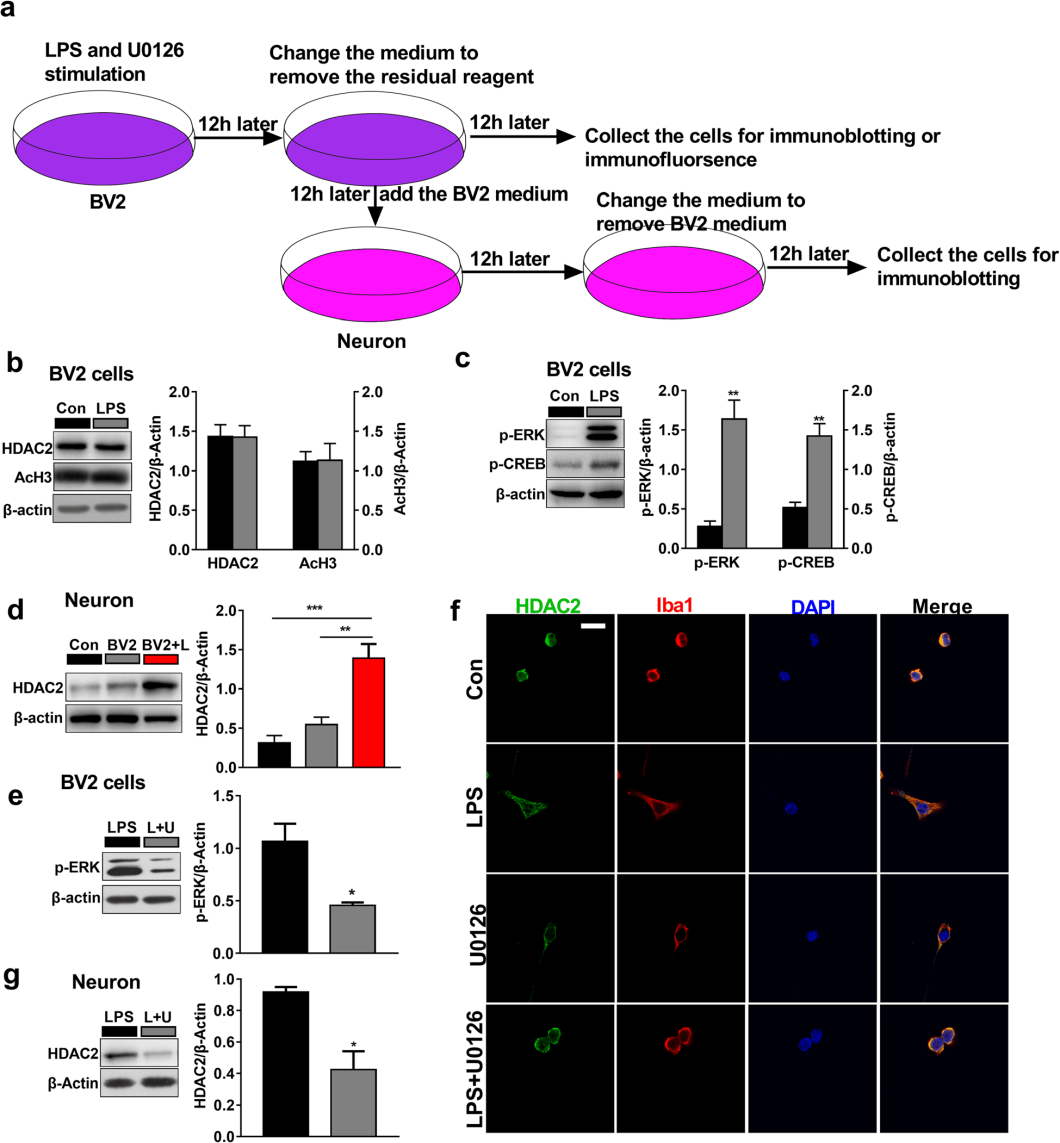
LPS induced microglial activation via the MAPK pathway, and the expression of neuronal HDAC2 is mediated by microglial activity. **a** Schematic diagram of the in vitro experiments. **b** The expression of HDAC2 and acetyl-H3 remained unchanged in the BV2 microglia in the LPS group (***n*** = 4). **c** The expression of p-ERK and p-CREB in BV2 microglia was increased in the LPS group (***n*** = 4). **d** The expression of HDAC2 in neurons was increased in the BV2 + LPS group compared with the origin neuron medium group (***n*** = 4). Con represent the origin neuron medium, BV2 represents the BV2 microglia untreated medium group and BV2 + L represents the BV2 treated with LPS group. **e** Compared with the LPS treatment, the ERK inhibitor U0126 significantly decreased the expression of p-ERK in neurons (***n*** = 3). U represents U0126. **f** The LPS-induced activation of microglia was significantly reversed by U0126. U represents U0126. **g** Compared with the LPS treatment, the ERK inhibitor U0126 significantly decreased the expression of HDAC2 in neurons (***n*** = 3). Data are presented as the mean ± S.E.M. ^*^P < 0.05; ^**^P < 0.01; and ^***^P < 0.001

In vitro experiments were performed to further explore the cellular and molecular mechanisms of LPS in cultured BV2 microglia cells and primary hippocampal neurons (Fig. 4a). As expected, our data showed that the levels of p-ERK1/2 (*t*_(3, 0.05)_ = 5.485, *P* < 0.01) and p-CREB (*t*_(3, 0.05)_ = 5.288 *P* < 0.01; Fig. 4b) were significantly increased upon LPS (1 μg/ml) in cultured BV2 microglia. The expressions of HDAC2 (*t*_(3, 0.05)_ = 0.04447, *P* = 0.966) and acetyl-H3 (*t*_(3, 0.05)_ = 0.05584, *P* = 0.9573; Fig. 4c) showed no significant changes. Next, we transferred the culture medium from microglia treated with LPS or left untreated (which had been changed to remove residual LPS) to cultured hippocampal neurons and observed that the expression of HDAC2 increased (*F*(2.9) = 19.26, *P* < 0.001; Fig. 4d) in the LPS treated BV2 medium group compared with the original neuron medium group. There seemed to be an increase of the expression of HDAC2 in the BV2 untreated medium group, but the difference was not statistically significant (*P* = 0.4426). We then pharmacologically inhibited the ERK pathway with U0126 (10 μmol/ml, the medium was also changed to remove residual U0126). We found that the level of p-ERK was decreased in the U0126 group compared with the control group (*t*_(2, 0.05)_ = 3.665, *P* < 0.05; Fig. 4e). The activation of microglia was reversed compared with LPS treatment (*t*_(2, 0.05)_ = 3.665, *P* < 0.05; Fig. 4f). HDAC2 was significantly repressed in primary neurons when microglial activity was repressed with U0126 (*t*_(2, 0.05)_ = 4.084, *P* < 0.05; Fig. 4 g). These results suggest that microglial activation mediate HDAC2 hyperexpression in hippocampal glutamatergic neurons.

### LPS represses BDNF and c-Fos expression in hippocampal glutamatergic neurons

**Fig. 5:**
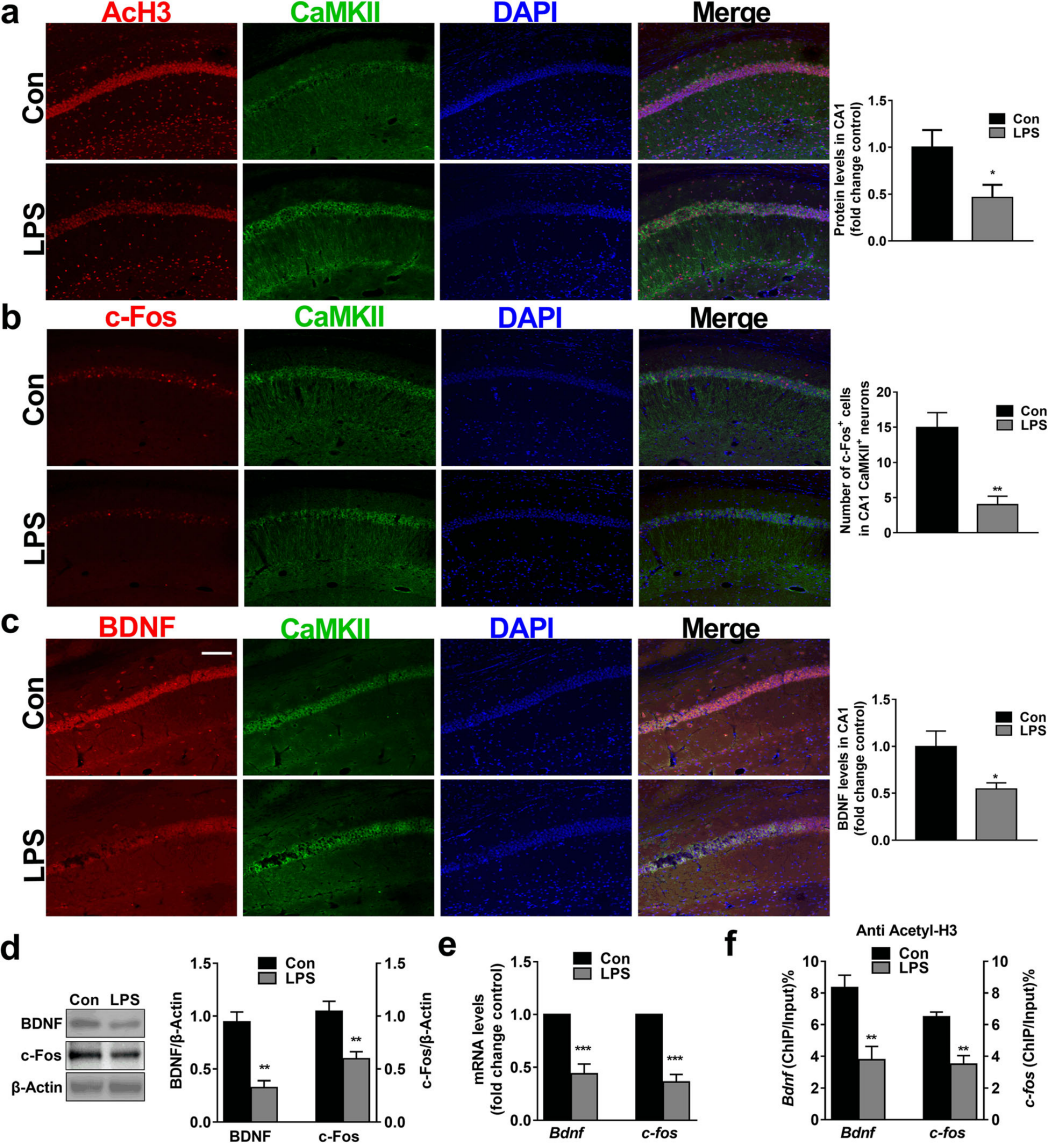
LPS decreased the expression of acetyl-H3, BDNF and c-Fos and suppressed *Bdnf* and *c-fos* transcription in hippocampal CaMKII^+^ neurons. **a** The levels of acetyl-H3 were decreased in the LPS group (*n* = 4 sections from 4 mice each, scale bar = 100 μm). **b** LPS exposure significantly decreased the expression of BDNF in CaMKII^+^ neurons in the LPS group (*n* = 4 sections from 4 mice each, scale bar = 100 μm). **c** The numbers of c-Fos colocalized with CaMKII neurons significantly decreased in the hippocampal CA1 region in the LPS group (*n* = 4 sections from 4 mice each, scale bar = 100 μm). **d** The levels of BDNF and c-Fos were decreased in the LPS group (*n* = 4). **e** The mRNA expression of *Bdnf* and *c-fos* was decreased in the LPS group (*n* = 4). **f** The levels of *Bdnf* and *c-fos*, which were bound by acetyl-H3, were decreased in the LPS group. Data are presented as the mean ± S.E.M. ^*^P < 0.05; ^**^P < 0.01; and ^***^P < 0.001

Various genes are regulated by histone acetylation, such as *Bdnf* and *c-fos*, which are involved in learning and memory [51–59]. Most neurons distributed in the hippocampus are glutamatergic and contain abundant BDNF and c-Fos [60–63]. We found that the majorities of Acetyl-H3, BDNF and c-Fos were expressed on CaMKII^+^ neurons and that LPS significantly decreased the levels of Acetyl-H3 (*t*_(3,0.05)_ = 2.727, *P* < 0.05), BDNF (*t*_(3, 0.05)_ = 2.569, *P* < 0.05) and c-Fos (*t*_(3, 0.05)_ = 4.392, *P* < 0.01) in hippocampal CaMKII^+^ neurons (Fig. 5a–c). We also found that the protein expressions of hippocampal BDNF (*t*_(3, 0.05)_ = 5.413, *P* < 0.01) and c-Fos (*t*_(3, 0.05)_ = 3.944, *P* < 0.01; Fig. 5d), as well as the mRNA expressions of *Bdnf* (*t*_(3, 0.05)_ = 6.091, *P* < 0.001) and *c-fos* (*t*_(3, 0.05)_ = 8.995, *P* < 0.001; Fig. 5e) were significantly decreased in the LPS-treated mice compared with the control mice. Histone acetylation contributes to an early step in the process of chromatin modification by disassembling nucleosomes to make DNA promoter regions accessible for transcription factor binding [64]. To further investigate whether histone deacetylation was involved in decreasing *Bdnf* and *c-fos* mRNA levels, we performed a chromatin immunoprecipitation assay (ChIP) with an anti-acetyl-H3 antibody. Our data showed that the levels of both *Bdnf* (*t*_(3, 0.05)_ = 53.948, *P* < 0.01) and *c-fos* (*t*_(3, 0.05)_ = 4.818, *P* < 0.01; Fig. 5f), which bonded to acetyl-H3, were decreased, making the *Bdnf* and *c-fos* promoter regions less accessible for transcription factor binding. The hypoexpression of BDNF and c-Fos in glutamatergic neurons induced by LPS is closely related to HDAC2.

### Suppressing HDAC2 in the hippocampal glutamatergic neurons reverses microglial activity, rescues BDNF and c-Fos expressions induced by LPS

**Fig. 6:**
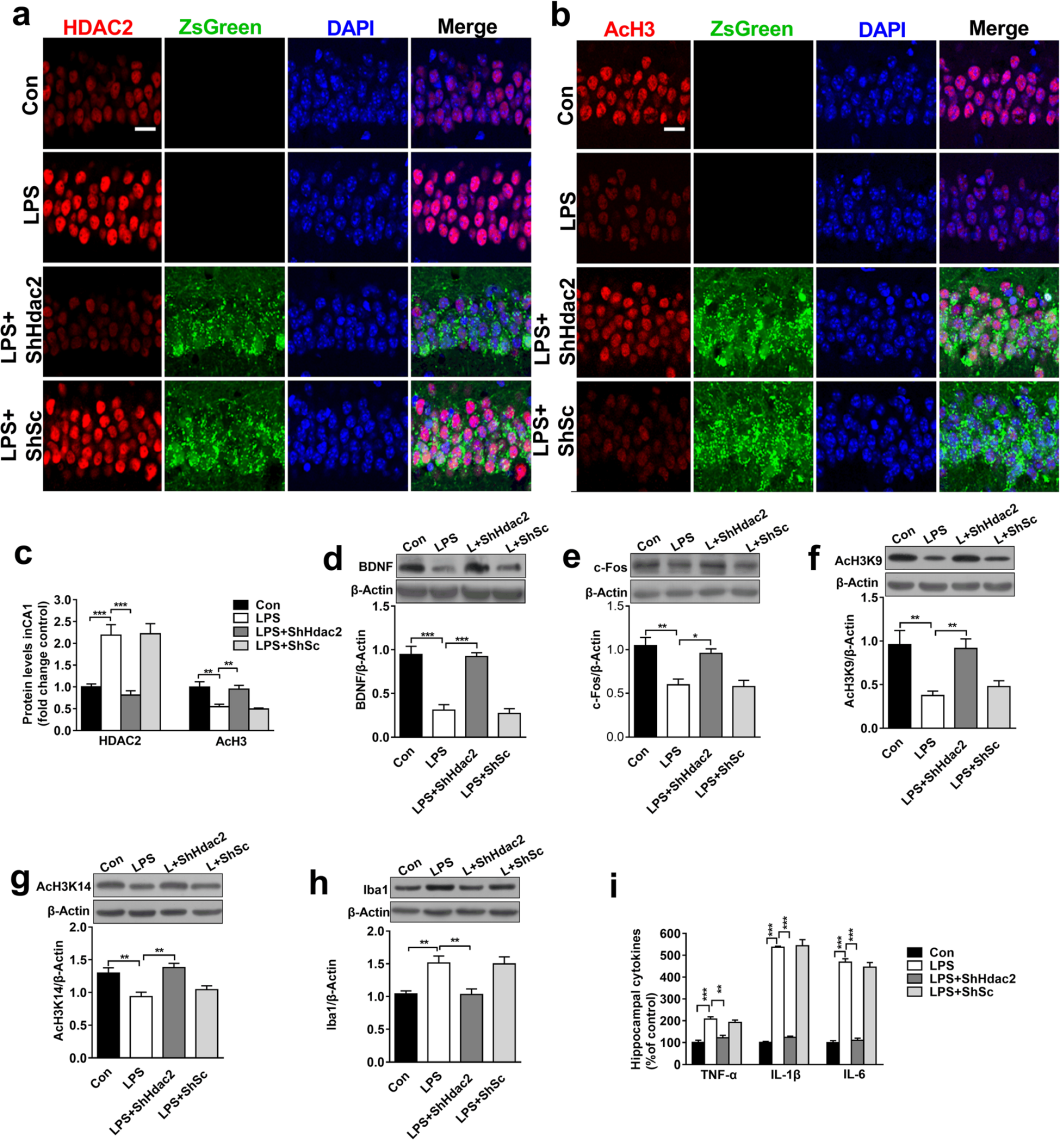
Suppression of HDAC2 reversed the alterations in BDNF and c-Fos and microglial activation induced by LPS. **a–b** Representative immunofluorescence images depicting HDAC2 and acetyl-H3 expression in the hippocampal CA1 region in mice treated with ShHdac2-ZsGreen are shown (*n* = 4, scale bar = 50 μm). **c** Suppressing HDAC2 expression reversed the LPS-associated decreases in HDAC2 and acetyl-H3 expression in the dorsal hippocampal CA1 region (*n* = 4 sections from 4 mice each). **d–g** Suppressing HDAC2 expression reversed LPS-induced alterations in BDNF, c-Fos, acetyl-H3K9, and acetyl-H3K14 expression in the dorsal hippocampal CA1 region (*n* = 4). **h** Suppressing HDAC2 expression reversed Iba1 alterations in the LPS group. **i** The levels of TNF-α, IL-1 and IL-6 in samples from the hippocampus were increased in the LPS + ShHdac2 group compared with the LPS group (*n* = 4). Data are presented as the mean ± S.E. M. ^*^P < 0.05; ^**^P < 0.01; and ^***^P < 0.001

To specifically confirm the role of HDAC2 in memory deficits, ShHdac2 was injected bilaterally into the dorsal hippocampus to suppress HDAC2. The expression of HDAC2 was inhibited by ShHdac2 (*F*_(3,12)_ = 17.95, *P* < 0.001). This suppression also reversed the levels of acetyl-H3 (*F*_(3,12)_ = 10.902, *P* < 0.01), BDNF (*F*_(3,12)_ = 31.565, *P* < 0.001), c-Fos (*F*_(3,12)_ = 11.259, *P* < 0.01), acetyl-H3K9 (*F*_(3,12)_ = 7.803, *P* < 0.01) and acetyl-H3K14 (*F*_(3,12)_ = 9.462, *P* < 0.01) in the LPS + ShHdac2 group compared with the LPS group (Fig. 6c–g). There is no significant difference between the LPS + ShSc group and the Con group. Moreover, we also observed that compared with LPS treatment, the suppression of HDAC2 in the hippocampus with ShHdac2 treatment decreased the high expressions of Iba1 (*F*_(3,12)_ = 8.866, *P* < 0.01, Fig. 6h) and microglia-secreted TNF-α (*t*_(3, 0.05)_ = 5.395, *P* < 0.01), IL-1β (*t*_(3, 0.05)_ = 14.93, *P* < 0.001) and IL-6 in the hippocampus (*t*_(3, 0.05)_ = 14.4, *P* < 0.001; Fig. 6i). These results indicated that neurons were not only passive targets of microglia but rather players with an active role in regulating microglial activity. HDAC2 is a key factor regulating neuronal transcription and the cross-talk between neurons and microglia under hyperinflammatory conditions.

### Suppressing HDAC2 in the Hippocampal glutamatergic neurons rescues memory deficits induced by LPS

**Fig. 7:**
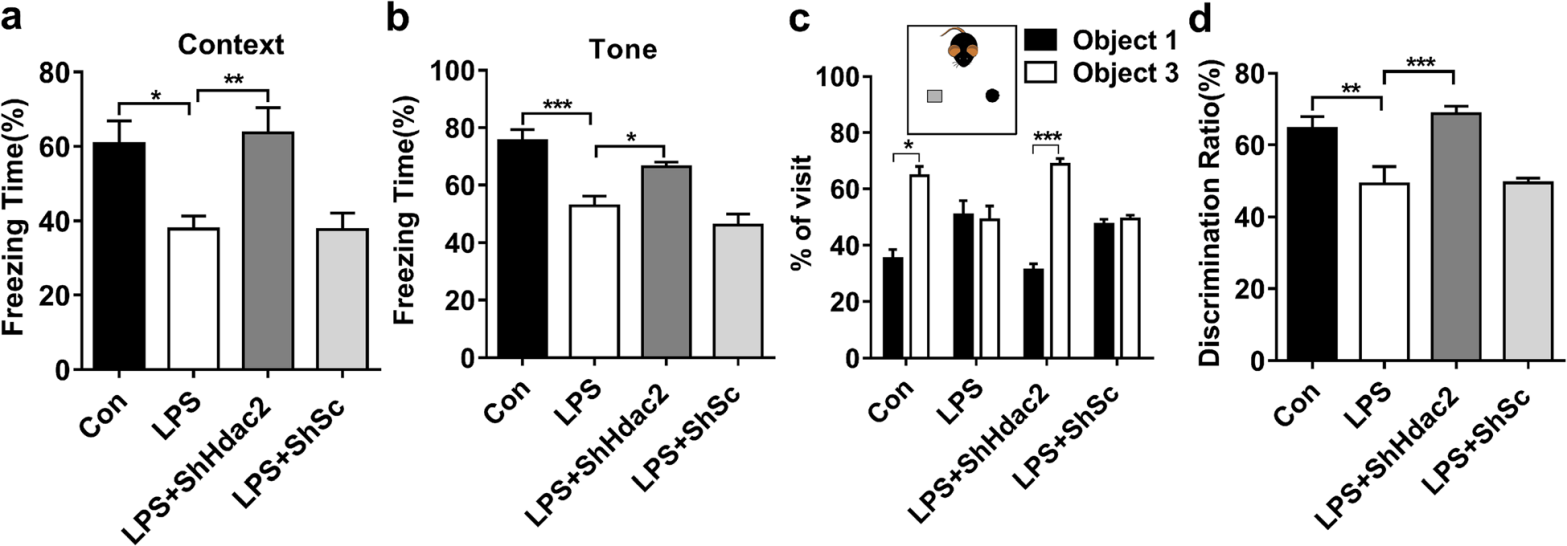
Suppression of HDAC2 restored cognitive dysfunction induced by LPS. **a**, **b** The suppression of HDAC2 expression significantly improved the impairments in context-dependent fear memory and tone-dependent fear memory observed after LPS administration (*n* = 10). **c** The suppression of HDAC2 expression significantly improved the impaired object discrimination observed after LPS administration, as revealed by similar percentages for visiting familiar object 1 and novel object 3 (*n* = 10). **d** Compared with those in the LPS group, the mice in the LPS + ShHdac2 group showed a preference for novel object 3 (*n* = 10). Data are presented as the mean ± S.E. M. ^*^P < 0.05; ^**^P < 0.01; and ^***^P < 0.001

Next we further investigated whether suppressing HDAC2 in the dorsal hippocampus would rescue the memory deficits induced by LPS. In the open-field test, the total travelled distance (Additional file 1: Figure S1f) and time spent on the centre area (Additional file 1: Figure S1 g) showed no significant differences among all groups. In the fear conditioning test, the freezing times both in the contextual and cued conditioning tests were reversed in the ShHdac2 + LPS group compared with the LPS group (*F*_(3,36)_ = 6.801, *P* < 0.01**;** Fig. 7a, *F*_(3,36)_ = 15.698, *P* < 0.001; Fig. 7b). In the novel object recognition task, all groups showed no significant preference in the novel objection recognition test during training (Additional file 1: Figure S1 h-i). During testing, the mice in the LPS group and LPS + ShSc group did not spend more time exploring the novel object than the exploring familiar object, whereas the mice in the LPS + ShHdac2 (*t*_(9, 0.05)_ = 9.17, *P* < 0.001) group and Con group (*t*_(9, 0.05)_ = 4.48, *P* < 0.05; Fig. 7c) did spend more time on exploring the novel object than the familiar object. The discrimination ratio was reversed by HDAC2 suppression compared with LPS treatment (*F*_(3,36)_ = 10.20, *P* < 0.001; Fig. 7d). On the basis of our present data, the impairment of memory performance induced by LPS exposure was restored by suppressing HDAC2.

## Discussion

### HDAC2 as a key regulator in anaesthesia- and inflammation-induced cognitive deficits

Accumulating evidence suggests that neuroinflammation and anaesthetics play critical roles in cognitive impairment [65–67]. However, the divergence in results from various studies demonstrates that the underlying mechanism needs to be further investigated [68–70]. In the present study, LPS-impaired object recognition and fear learning were not aggravated by ISO in adult mice, which suggested that a single use of ISO would not increase memory deficits in young patients with acute abdominal inflammation. However, clinical studies need to be further investigated.

We observed that LPS, but not ISO, increased the expression of HDAC2 in the hippocampus in adult mice. Previous studies demonstrated that cognitive impairment is highly related to HDAC2, and repeated exposure to ISO in aged rats upregulated HDAC2 expression [71]. Maternal exposure to isoflurane impaired learning and memory of the offspring and increased HDAC mRNA [72]. Age may be an independent susceptibility factor resulting in anaesthesia-induced cognitive impairment. Consistent with our finding that object recognition and fear memory were impaired in aged (> 18 months) mice exposed to ISO (data not shown). The discrepancies between the adult (3 months) and aged (> 18 months) mice exposed to ISO inhalation might result from the different ages. The general anesthetic may impair developing neurons and induce cognitive dysfunction in a dose- and time- dependent manner [73]. However, the effect of exposure of isoflurane on cognitive function remains contradictory. The results of ISO may due to the concentration, the times and the age of mice of the study design [72–78]. Several anesthetics have the similar properties with ISO. In vitro and mouse model studies suggest that propofol inhibits, while isoflurane increases neuroinflammation [79, 80]. Propofol may inhibit the neuroinflammatory response elicited by LPS and protect the brain from injury. The effect of other anaesthetics may be investigated in the future.

### The role of microglia in regulating neuronal HDAC2 expression

HDAC2 is known to participate in memory impairment, and hyperexpression of HDAC2 is involved in neurodegenerative disorders such as Alzheimer’s disease [81]. We found that the HDAC2 was mainly distributed in hippocampal glutamatergic neurons, which indicated that HDAC2 plays an important role in regulating histone acetylation and the transcription of neurons. As is known, LPS elicited inflammatory cytokines response in mice [82, 83]. Due to the role of HDAC2 in negatively regulating gene expression, the present study demonstrated that LPS induced pro-inflammatory cytokine transcription and microglial Iba1 expression in different ways. CREB is an important transcription factor participating in the activation of multiple immediate early genes. The hyperphosphorylation of CREB at Ser^133^ via MSK1/2 occurs in response to the activation of MAPK [57, 84]. In this study, we found that LPS induced the activation of microglia and increased the expression of pro-inflammatory cytokines through the MAPK pathway.

It is well documented that activated microglia release several chemokines, such as neurotrophic factors, affecting neuronal survival and pro-inflammatory mediators [85, 86]. Previous studies have described signals from activated microglia to neurons. We also found that inactivation of BV2 microglia reversed the hyperexpression of HDAC2 in hippocampal neurons. All the results provided evidence that the activation of microglia negatively affected the transcription of neurons.

### HDAC2 mediates microglial activation and negatively regulates BDNF and c-Fos expression as well as cognitive function

The histone modification is involved in the regulation of BDNF and c-Fos and in learning and memory. LPS reduced *Bndf* and *c-fos* transcription as well as the mRNA levels of *Bdnf* and *c-fos*, which subsequently decreased the protein levels of BDNF and c-Fos in hippocampal glutamatergic neurons. The LPS-induced decrease in BDNF and c-Fos in glutamatergic neurons is closely related to HDAC2. ShHDAC2, instead of the microglia inhibitor minocycline [87, 88]. was chosen to specifically inhibit HDAC2. We observed that the suppression of HDAC2 expression reversed the expressions of BDNF and c-Fos as well as microglial activity in the LPS group. These data further support the role of HDAC2 in regulating microglial activity and gene transcription of *Bdnf* and *c-fos* in glutamatergic neurons. Previous study reported that HDAC2 expression has been shown to be protective in inflammatory conditions in RAW264.7 cell line. The discrepancy of HDAC functions may due to their expression in different cell lines [89]. On the basis of currently available studies, HDAC2 expression in hippocampal glutamatergic neurons is important for memory in a hyperinflammatory state. Our investigation reveals a previously unrecognized cell-specific role of HDAC2 in regulating the transcriptional activity of hippocampal glutamatergic neurons and in the interaction of neurons and microglia. Current commercially available BBB-permeable HDAC inhibitors used so far exhibit little isoform-specificity (i.e. pan-HDAC inhibitors) [90]. The development of new specific BBB-permeable HDAC2 inhibitor may be a new therapeutic approach for neuroinflammation treatments. A variety of further investigations are needed in the future, such as determining which kinds of receptors on neurons are affected by neuroinflammation and which pathways and potential transmitter-receptor interactions regulate glial activation.

## Conclusions

In summary, our findings consistently demonstrated that microglial activation increased HDAC2 expression leading to *Bdnf* and *c-fos* transcription repression in hippocampal glutamatergic neurons, which resulted in memory impairment. Then, HDAC2 repression in hippocampal glutamatergic neurons rescued the expressions of BDNF and c-Fos and reversed microglial activation, which prevented cognitive dysfunction in mice. Neurons are not merely passive targets of microglia but play an active role in regulating microglial activity. The present study revealed a novel mechanism for HDAC2-mediated modulation of cognitive dysfunction in the hippocampus. The findings of the research also provided a potential research direction and promising strategy for avoiding cognitive deficits in the clinic.

## Supplementary information

**Additional file 1: Figure S1.** Timeline of the procedures for all the in vivo experiments and the effect of LPS and ISO or ShHdac2 on emotional behaviour in mice. **a** Timeline of the procedures for all the in vivo experiments. **b** The mice showed normal total travelled distances in the open-field test (*n*=8). **c** The mice showed normal amounts of time spent in the centre area (*n*=8). **d-e** No preference for object 1 or object 2 and similar exploration of object 2 were observed in all groups during training in the novel object recognition task (*n*=8). **f** The mice showed normal total travelled distances in the open-field test (*n* = 10). **g** The mice showed normal amounts of time spent in the centre area in the open-field test in the LPS + ShHdac2 group compared to the LPS group (*n*=10). **h-i** No preference for object 1 or object 2 and similar exploration of object 2 in all groups were observed during training in the novel object recognition task (*n*=10). Data are presented as the mean ± S.E.M. ^*^P < 0.05; ^**^P < 0.01; and ^***^P < 0.001.

**Additional file 2: Figure S2.** Schematic diagram of the injector cannula tips used in the experiments shown in the panel and Nissl staining illustrating the intra-hippocampal injection site in the mouse brain. **a** Nissl staining illustrating the intra-hippocampal injection site (scale bar=200 μm). **b** Schematic diagram of the injector cannula tips.

**Additional file 3: Figure S3.** Isoflurane did not induce Iba1 expression and pro-inflammatory responses in the periphery and hippocampus. **a-b** Immunostaining and immunoblotting for Iba1 in the hippocampal CA1 region remained unchanged in the ISO group compared to the Con group (*n* = 3-4 sections from 4 mice, scale bar, 200 μm). **c** The levels of TNF-α, IL-1β, and IL-6 in plasma samples showed no significant changes in the ISO group compared to the Con group (*n*=4). **d** The protein levels of TNF-α, IL-1β and IL-6 in samples from the hippocampus remained unchanged in the ISO group (*n*=4). Data are presented as the mean ± S.E.M. ^*^P < 0.05; ^**^P < 0.01; and ^***^P < 0.001.

**Additional file 4: Figure S4.** The cellular morphology of astrocyte remained nearly unaffected after LPS administration. Immunofluorescent staining of GFAP as marker for astrocytes in hippocampus of LPS treated and Con group mice (3-4 sections from 4 mice, scale bar=10 μm).

## Data Availability

The datasets during and/or analysed during the current study are available from the corresponding author on reasonable request.

## Abbreviations

AAV: Adeno-associated virus
AD: Alzheimer’s disease
BDNF: Brain-derived neurotrophic factor
BSA: Bovine serum albumin
CaMKII: Calcium/calmodulin-dependent protein kinases
ChIP-qPCR: Chromatin immunoprecipitation quantitative real-time reverse transcription PCR
CREB: cAMP-response element-binding protein
DAPI: 4′,6-diamidino-2-phenylindole
ECL: Enhanced chemiluminescence
ELISA: Enzyme-linked immunosorbent assay
ERK 1/2: Extracellular signal-regulated protein kinase 1/2
GFAP: Glial fibrillary acidic protein
HATs: Histone acetyltransferases
HDACs: Histone deacetylases
MAC: Minimum alveolar concentration
Iba1: Ionized calcium-binding adaptor molecule1
IL-1β: interleukin-1β
IL-6: interleukin-6
ISO: isoflurane
LPS: Lipopolysaccharide
S.E.M.: Standard error of the mean
ShHdac2: pAAV-ZsGreen-ShRNA-mHDAC2
ShSc: pAAV-ZsGreen-ShRNA-mScramble
TLR4: Toll-like receptor 4
TNF-α: tumour necrosis factor α
ZsGreen: *Zoanthus* green fluorescent protein
