# Supplementary figures and images for: HDAC2 hyperexpression alters hippocampal neuronal transcription and microglial activity in neuroinflammation-induced cognitive dysfunction
Source: J Neuroinflammation. 2019 Dec 3;16:249. doi: 10.1186/s12974-019-1640-z (PMC6889553; doi:10.1186/s12974-019-1640-z)

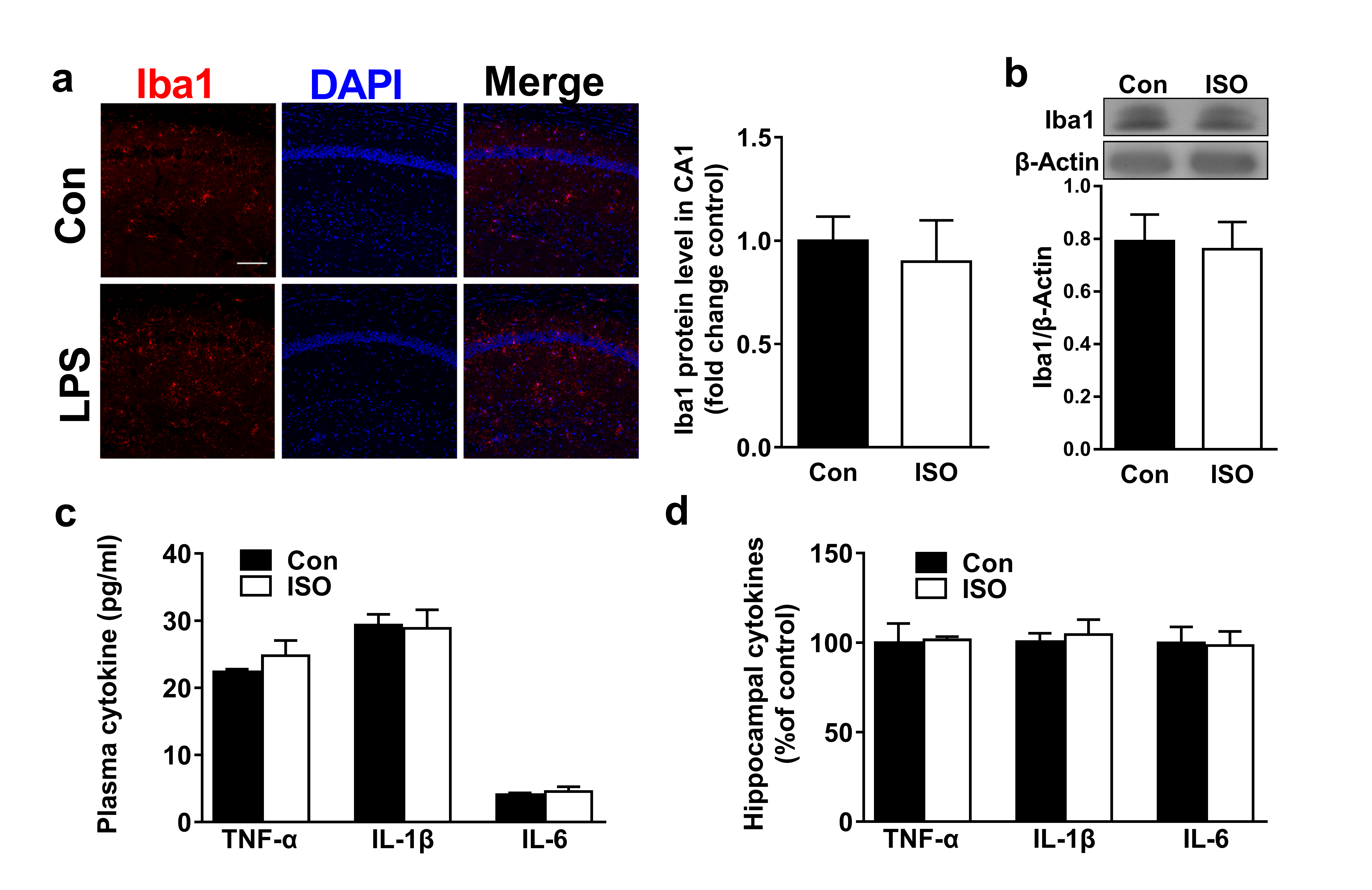

Supplement: Supplementary file 3 — Additional file 3: Figure S3. Isoflurane did not induce Iba1 expression and pro-inflammatory responses in the periphery and hippocampus. a-b Immunostaining and immunoblotting for Iba1 in the hippocampal CA1 region remained unchanged in the ISO group compared to the Con group (n = 3-4 sections from 4 mice, scale bar, 200 μm). c The levels of TNF-α, IL-1β, and IL-6 in plasma samples showed no significant changes in the ISO group compared to the Con group (n=4). d The protein levels of TNF-α, IL-1β and IL-6 in samples from the hippocampus remained unchanged in the ISO group (n=4). Data are presented as the mean ± S.E.M. *P < 0.05; **P < 0.01; and ***P < 0.001. [file 12974_2019_1640_MOESM3_ESM.tif]

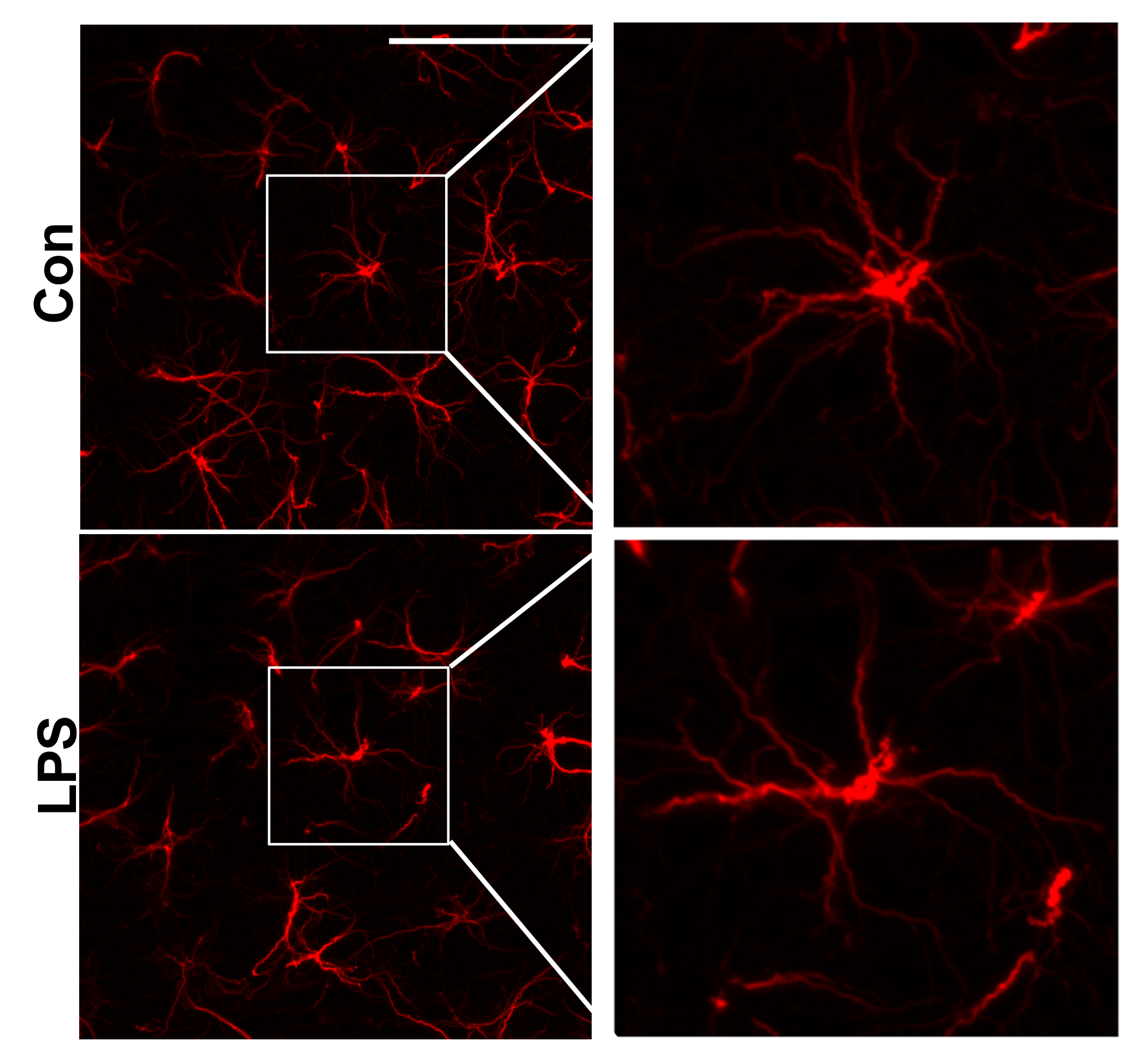

Supplement: Supplementary file 4 — Additional file 4: Figure S4. The cellular morphology of astrocyte remained nearly unaffected after LPS administration. Immunofluorescent staining of GFAP as marker for astrocytes in hippocampus of LPS treated and Con group mice (3-4 sections from 4 mice, scale bar=10 μm). [file 12974_2019_1640_MOESM4_ESM.tif]
